# Anesthetic management of a patient with sodium-channel myotonia: a case report

**DOI:** 10.1186/s40981-019-0300-8

**Published:** 2019-11-25

**Authors:** Naohisa Matsumoto, Rei Nishimoto, Yoshikazu Matsuoka, Yoshimasa Takeda, Hiroshi Morimatsu

**Affiliations:** Department of Anesthesiology and Resuscitology, Okayama University Hospital, 2-5-1 Shikata-cho, kita-ku, Okayama City, Okayama 700-8558 Japan

**Keywords:** Anesthetic management, Myotonia congenita, Nondystrophic myotonia, Paramyotonia congenita, Periodic paralysis, Potassium-aggravated myotonia, Skeletal muscle channelopathy, Sodium-channel myotonia

## Abstract

**Background:**

Sodium-channel myotonia (SCM) is a nondystrophic myotonia, characterized by pure myotonia without muscle weakness or paramyotonia. The prevalence of skeletal muscle channelopathies is approximately 1 in 100,000, and the prevalence of SCM is much lower. To our knowledge, this is the first report on anesthetic management of a patient with SCM.

**Case presentation:**

A 23-year-old woman with congenital nasal dysplasia and SCM was scheduled to undergo rhinoplasty with autologous costal cartilage. Total intravenous anesthesia without muscle relaxants was administered followed by continuous intercostal nerve block. Although transient elevation of potassium level in the blood was observed during surgery, the patient did not show exacerbation of myotonic or paralytic symptoms in the postoperative period.

**Conclusion:**

Total intravenous anesthesia and peripheral nerve block can be administered safely to a patient with SCM. However, careful monitoring of the symptoms and electrolytes is recommended.

## Background

Sodium-channel myotonia (SCM) belongs to a group of nondystrophic myotonias and is caused by mutations in the sodium-channel gene on chromosome 17q, encoding the α-subunit protein of the voltage-gated sodium channel Nav1.4 (*SCN4A*) expressed in the skeletal muscle [1]. It is characterized by pure myotonia, without muscle weakness or paramyotonia, and is triggered by exercise and the ingestion of potassium-rich food [1]. In patients with SCM, special attention must be paid to the choice of anesthetic agents, surgical pain management, and body temperature management as all of these affect muscle contraction. Because the prevalence of skeletal muscle channelopathies is approximately 1 in 100,000 and that of SCM is much less [2], little is known about how to safely administer anesthesia to these patients [3]. In fact, our literature search failed to yield any clinical reports on the anesthetic management of patients with SCM. Herein, we report the anesthetic management of a patient diagnosed with SCM.

## Case presentation

The patient was a 23-year-old woman (height 147.6 cm, weight 43.5 kg) with congenital nasal dysplasia (Binder syndrome). At the age of 3 years, the patient struggled to initiate walking and to climb the stairs; additionally, she exhibited grip myotonia and struggled to open her eyes and mouth after sneezing. Exercise and cold exposure induced symptom exacerbation. The patient’s mother and younger sister also exhibited similar symptoms. The patient was referred to our hospital, but after physical examination, she was not definitively diagnosed with a myotonic syndrome. She was followed up. At the age of 8 years, the patient underwent rhinoplasty with iliac bone graft under general anesthesia without complications. At the age of 10 years, the patient underwent posterior cervical fixation (C1–2) for traumatic atlantoaxial subluxation under general anesthesia. However, when the patient was extubated following surgery, she exhibited acute dyspnea in the operating room and was immediately re-intubated and transferred to the intensive care unit. After discharge, the patient visited our hospital again and genetic analysis revealed a mutation at V445M in the *SCN4A* gene, leading to a diagnosis of SCM. As the patient grew, the nasal deformity became conspicuous with increasing atrophy of the graft. Therefore, the patient was scheduled to undergo a second rhinoplasty with autologous costal cartilage.

Pre-operative test results, including those of blood screening, blood gas analysis, X-ray examination, respiratory function test, and electrocardiogram, were within normal limits. The patient’s SCM-related symptoms were stable. Mexiletine and eperisone were taken as needed. General anesthesia was induced with target-controlled infusion of propofol (5 μg mL^− 1^), continuous infusion of remifentanil (0.3 μg kg^− 1^ min^− 1^), and fentanyl (200 μg), without any muscle relaxants. Due to the posterior fixation of the patient’s cervical spine, a McGrath® video laryngoscope (Covidien, Japan) was used. Orotracheal intubation was performed without inducing a cough reflex. Anesthesia was maintained with propofol (2–2.4 μg mL^− 1^), remifentanil (0.1–0.25 μg kg^− 1^ min^− 1^), and fentanyl (300 μg). Arterial blood pressure and BIS® index (Covidien, Japan) were monitored in addition to standard monitoring. Body temperature at the bladder was maintained between 36.0 and 37.4 °C with a forced-air warming system.

**Fig. 1:**
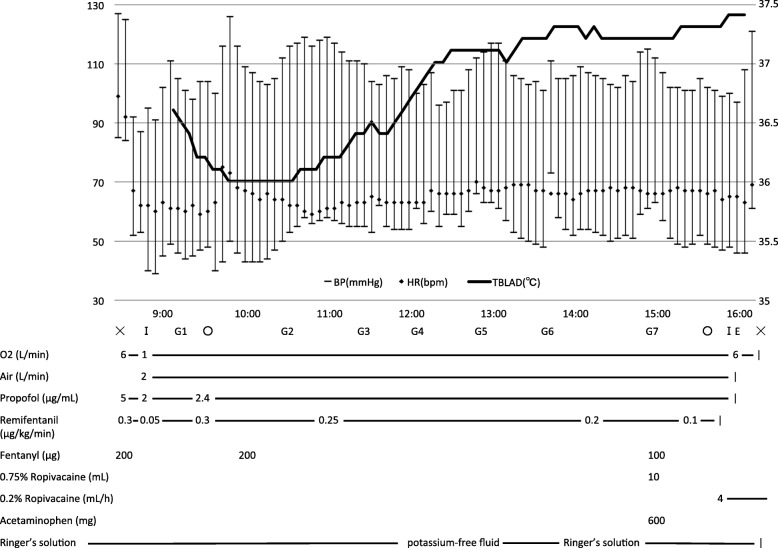
Anesthesia record of the present case. BP, blood pressure (left axis); E, extubation; G1–7, the time points of arterial blood gas analysis; HR, heart rate (left axis); I, intubation; TBLAD, urinary bladder temperature (right axis); ×, start and end of the anesthesia; ○, start and end of the surgery

As shown in Fig. 1 and Table 1, the blood concentration of potassium ion rose during general anesthesia. After changing Ringer’s solution to a potassium-free fluid, the blood concentration of potassium ion returned to within the normal range without any additional therapeutic intervention. For postoperative analgesia, intercostal nerve block with 10 mL of 0.75% ropivacaine was administered through a catheter (Perifix® ONE catheter, B. Braun, Japan). Continuous infusion of 0.2% ropivacaine was started at 4 mL/h after the operation. Intraoperative cardiovascular and respiratory vital signs remained stable. After the operation was concluded, we exchanged the orotracheal tube for a supraglottic airway device (Air-Q™ #3.5, Intermed Japan, Japan). The supraglottic airway device was removed uneventfully after we confirmed that the patient had regained consciousness and initiated spontaneous breathing. The patient did not exhibit shivering or exacerbation of other symptoms related to SCM. The patient was transferred to the intensive care unit overnight and was discharged from the hospital on postoperative day 7.

**Table 1:** Results of arterial blood gas analysis

	Pre-op	G1	G2	G3	G4	G5	G6	G7	Post-op
pH	7.41	7.41	7.40	7.43	7.45	7.41	7.42	7.43	7.41
pCO_2_ (mmHg)	41.1	35.9	36.1	33.7	31.6	35.0	34.9	35.1	38.2
pO_2_ (mmHg)	100	334	273	274	239	273	267	265	132
HCO_3_^−^ (mEq/L)	25.5	22.2	21.8	22.0	21.4	22.0	22.5	23.0	23.7
BE (mEq/L)	1.3	−1.5	−2.0	−1.2	− 1.5	−1.6	− 1.0	− 0.4	− 0.3
Hb (g/dL)	12.3	11.1	11.0	11.3	11.3	11.1	10.6	10.6	11.6
Glu (mg/dL)	NA	109	125	99	98	128	112	84	89
Na^+^ (mEq/L)	140	139	142	141	142	141	143	144	137
K^+^ (mEq/L)	3.8	3.5	3.4	5.2	5.4	4.9	3.5	3.1	3.4
Cl^−^ (mEq/L)	107	109	111	113	116	114	115	115	108
Ca^2+^ (mEq/L)	1.21	1.15	1.15	1.17	1.15	1.16	1.14	1.16	1.16

*BE* base excess. G1–7: time points of these analyses are shown in Fig. 1

## Discussion

Skeletal muscle channelopathies are divided into periodic paralyses and nondystrophic myotonias [1]. Nondystrophic myotonia is characterized by muscle stiffness on voluntary movement owing to delayed skeletal muscle relaxation. Nondystrophic myotonias include myotonia congenita, paramyotonia congenita, and SCM. Because SCM is very rare, there have been no reports describing perioperative anesthetic management of patients with SCM to date. For guidance, we referred to reports on anesthetic management of patients with myotonic dystrophy and other types of skeletal muscle channelopathies.

First, the anesthetic to be used was considered. Previous reports on the anesthetic management of patients with myotonic dystrophy and myotonia congenita [4–7] suggest that propofol can be used safely for induction and maintenance of general anesthesia in patients with SCM. Volatile agents, including sevoflurane and desflurane, may also be safe for use in patients with SCM because they are not contraindicated in patients with myotonic dystrophy and other myotonic diseases [3, 7, 8]. However, depolarizing muscle relaxants must be avoided because they may cause exaggerated contracture, masseter spasm, and laryngospasm, thus complicating extubation [3, 7]. The use of non-depolarizing muscle relaxants may be acceptable with monitoring of neuromuscular blockade [4], although the use of cholinesterase inhibitors might worsen the symptoms of SCM as it does in other myopathies. In the present case, we avoided muscle relaxants because we anticipated that the patient’s rhinoplasty and potential exacerbation of myotonia would independently increase the difficulty of her airway postoperatively.

An increase of serum potassium was noted during anesthesia, which might result from the potassium-containing solution and its redistribution [9]. It was rapidly decreased after switching it to a potassium-free solution. The mutation at V445M causes some alternations in the gating mechanism of NaV1.4 as impairment of fast inactivation and enhanced slow inactivation result in increased excitability of the muscles [10]. Additionally, pain or hypothermia can cause muscle contraction with excessive sodium influx in patients with SCM, which may result in an elevation of the extracellular potassium level. Thus, repeated intraoperative arterial blood gas analysis is recommended.

We made a concerted effort to prevent our patient from shivering, because shivering can exacerbate symptoms of myotonia [4]. This required particular focus on body temperature control and pain management. We rewarmed the patient and maintained her body temperature using a forced-air warming device throughout the surgery. To control pain, we administered intravenous fentanyl with a continuous intercostal nerve block, bearing in mind that excessive fentanyl during anesthesia should be avoided for patients with difficult airways. Combined use of regional anesthesia should be considered, if possible, in order to reduce opioid dose.

Oral administration of mexiletine might be used if the patient had an acute myotonic episode, because it is generally regarded as the only effective treatment for myotonic episodes [11]. It is a non-selective sodium channel inhibitor and belongs to the Vaughan-Williams class IB anti-arrhythmic group of drugs [12]. Although IB and IC anti-arrhythmic group of drugs have been studied, no other drug has been shown effective for treatment of myotonia. Additionally, the effectiveness of intravenous mexiletine is unknown. Usually myotonia lasts for seconds to minutes and improves with time; however, sometimes it lasts longer and involves respiratory and/or swallowing muscles. Therefore, we need to observe the patient carefully for signs of deterioration postoperatively [13]. Intensive treatment, including constant monitoring, is definitely required for patients with SCM after general anesthesia. The patient should be followed up for several hours after extubation in a postanesthesia care unit [14]. In addition, we need to provide preoperative education to the patient to enable self-recognition of worsening myotonia and notify the medical staff for help.

The present patient had a history of re-intubation following postoperative respiratory failure. For this reason, we decided to exchange the device used for airway maintenance as described above. Supraglottic airway devices allow cautious observation of respiratory conditions without tracheal stimulation [15], which can trigger myotonia in patients with SCM. This procedure should be considered for patients requiring tracheal intubation.

In conclusion, we safely administered total intravenous anesthesia to a patient with SCM, although the current literature does not present contraindications to the use of inhaled anesthetics in these patients. Given the clinical course of the present patient, close monitoring of electrolytes, airway patency, and symptoms is recommended.

## Data Availability

Not applicable

## Abbreviations

SCM: Sodium-channel myotonia
*SCN4A*: α-Subunit protein of the voltage-gated sodium channel Nav1.4
